# Root Surface Temperature Increases during Root Canal Filling In Vitro with Nd:YAG Laser-Softened Gutta-Percha

**DOI:** 10.1155/2020/8828272

**Published:** 2020-06-05

**Authors:** Błażej Podolak, Alicja Nowicka, Krzysztof Woźniak, Liliana Szyszka-Sommerfeld, Włodzimierz Dura, Mariusz Borawski, Elżbieta Dembowska, Mariusz Lipski

**Affiliations:** ^1^Private Practice, Kraków, Poland; ^2^Department of Conservative Dentistry and Endodontics, Pomeranian Medical University in Szczecin, Szczecin, Poland; ^3^Department of Orthodontics, Pomeranian Medical University in Szczecin, Szczecin, Poland; ^4^Department of Preclinical Conservative Dentistry and Preclinical Endodontics, Pomeranian Medical University in Szczecin, Szczecin, Poland; ^5^Faculty of Computer Science and Information Technology, West Pomeranian University of Technology, Szczecin, Poland; ^6^Department of Periodontology, Pomeranian Medical University in Szczecin, Szczecin, Poland

## Abstract

The aim of this in vitro study was to measure the temperature increases produced on the mesial and vestibular root surfaces of premolar teeth during a laser-softened gutta-percha obturation technique. We studied 12 extracted human premolar teeth with a single canal. After root canal cleaning and shaping, the teeth were obturated with gutta-percha that had been softened with a neodymium-doped yttrium aluminium garnet (Nd:YAG) laser (CTL 1503) at a wavelength of 1.064 nm. The laser setup parameters included a 30 Hz frequency and a 200 mJ/pulse with optical fiber tips of 0.320 mm diameter. A sectional warm gutta-percha condensation was used. Temperature changes on the whole mesial and vestibular outer surfaces of the roots were measured at approximately 2 s intervals with an infrared thermal imaging camera. A significantly higher increase in temperature was observed for the mesial root surface (7.5°C) compared to the vestibular surface (3.7°C) (*p* ≈ 0). The findings suggested that root canal filling with Nd:YAG laser-softened gutta-percha in premolar teeth is not likely to damage the surrounding periradicular tissues. To obtain valid temperature results, the measurement should be performed on the surface with the thinnest root wall.

## 1. Introduction

The success of conservative endodontic treatment depends on completely filling the root canal space with dimensionally stable, biologically compatible material [1]. Among the many materials that have been used to obturate the root canal space, gutta-percha is the most widely used, due to its inertness, biocompatibility, plasticity when warmed, and easy removal for post- or retreatments [2].

Cold lateral condensation of gutta-percha has proven to be a very popular and clinically effective obturation technique [3]. However, it has some inadequacies; for example, the accessory points sometimes separate from the master point, which creates voids, and, in the event of excessive condensation forces, it may result in vertical root fractures [4].

In order to avoid those problems, several warm gutta-percha methods have been introduced. These include warm vertical condensation [5], thermomechanical compaction [6], warm lateral condensation [7], high- and low-temperature thermoplasticized injectable gutta-percha [8, 9], and continuous wave of condensation technique [10]. Moreno [11] introduced the use of ultrasonically produced heat to soften gutta-percha. More recently, Anić and Matsumoto [12] used three different laser devices, including argon, CO_2_, and neodymium-doped yttrium aluminium garnet (Nd:YAG), as intracanal sources of heat for the sectional warm gutta-percha condensation technique. Among these lasers, the Nd:YAG laser is the most popular because a thin fiber-optic delivery system is available for entering narrow root canals with this device [12].

Even though tooth root tissues (dentin and cementum) have poor thermal conductivity [13], heat produced inside the lumen of the canal with a laser may partly radiate to the outer root surface and cause injury in the periodontal ligament and bone. Eriksson and Albrektsson [14] conducted a vital microscopic study to determine temperature threshold levels for heat-induced bone tissue injury in a rabbit model. A 10°C temperature increase for 1 min was found to cause reversible changes in the periodontal tissues of these animals. Bahcall et al. [15] reported that an intracanal irradiation with a Nd:YAG laser caused ankylosis, cemental lysis, and major bone remodeling 30 days after treatment (dog model). Thus, the primary concern in using a laser for root canal treatment is the potential deleterious effects on the structures surrounding the tooth root.

The aim of this in vitro study was to assess the temperature changes on the mesial and vestibular root surfaces of premolar teeth during Nd:YAG laser-softened gutta-percha obturation with the use of an infrared imaging camera.

## 2. Materials and Methods

### 2.1. Teeth Samples

We studied 12 human premolars with a single root canal that had been extracted for orthodontic and periodontal reasons. The roots were stripped of soft tissue and calculus with hand instruments. All specimens were microscopically inspected to find any defects or root fractures and to confirm the complete formation of apices. After access cavities were prepared and the pulp was extirpated, a size 10 K file was introduced into the canal until it slightly emerged from the apical foramen. The working length was established by subtracting 1 mm from this length. The canal was enlarged apically to size 40 with a K file. The apical one-third was flared with the step-back technique, and the middle and coronal two-thirds were shaped with sizes 2 through 4 Gates-Glidden drills. The canals were irrigated with 2 ml of 5.25% sodium hypochlorite solution after each instrument. Finally, the canals were flushed with 15% EDTA for 3 min, followed by irrigation with 5.25% sodium hypochlorite for 3 min with a syringe and needle, and dried with paper points.

### 2.2. Root Canal Obturation

**Figure 1:**
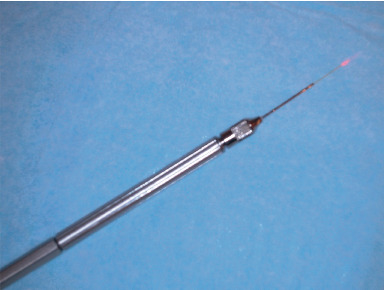
The optical fiber tips of 0.320 mm diameter (Nd:YAG laser—CTL-1503, Laserinstruments, Warsaw, Poland).

The teeth were obturated with the use of Nd:YAG laser—CTL-1503 (Laserinstruments, Warsaw, Poland)—at a wavelength of 1.064 nm. The setup parameters were 30 Hz frequency and a 200 mJ/pulse with optical fiber tips of 0.320 mm diameter (Figure 1). A sectional warm gutta-percha condensation technique was used, as described in [16]. The master gutta-percha cone was sectioned into 2 mm fragments. The tip fragment of the master cone was coated with an AH Plus root canal sealer (Dentsply/DeTrey, Munich, Germany) and inserted into the apical part of the root canal with a hand plugger (VDW, Munich, Germany). Next, the laser fiber was introduced into the canal, at 2 mm from the gutta-percha fragment. The gutta-percha was lased (4 s) and, after removal of the laser fiber, was condensed with a hand plugger. Next, a new fragment of gutta-percha was inserted into the canal, and the lasing and condensation were repeated. In total, this procedure was performed four times.

### 2.3. Temperature Measurements

To obtain root canal obturation and temperature measurements, the crowns of the teeth were fixed with the entire root surface exposed to the air. Temperature changes were recorded on the whole mesial root surfaces during root canal obturation with a ThermaCam SC500 thermal imaging camera (Flir, Danderyd, Sweden) with a focal plane array (FPA) of 320 × 240 pixels and an uncooled microbolometer detector. This camera had a spatial resolution of 1.3 mrad, a spectral range of 7.5–13 *μ*m, and a thermal sensitivity of 0.07°C at 30°C. The camera was mounted on a stand perpendicular to the root surface at a distance of 15 cm. The thermogram recording was initiated 2 s before root canal filling and continued at approximately 2 s intervals for a total of 90 s (for a precise interpretation of recorded thermograms, we applied the ThermaCAM Explorer 99 and ThermaCAM Reporter 2000 software packages). The experiment was carried out under controlled environmental conditions (ambient temperature = 25 ± 0.9°C, air flow < 0.5 m/s). The camera was calibrated for distance, ambient temperature, and emissivity of the root tissues. The emissivity of the root tissues was calculated to be 0.91, based on the method described in [17].

After the root canal obturation and temperature measurement, the fillings were removed with a set of Hedström files. Then, a second stage was performed, where the root canals were then filled in the same manner as in the first stage. The temperature was measured on the vestibular surface of the roots, instead of the mesial surface, in the same manner as in the first stage.

### 2.4. Statistical Analysis

The normal distribution was confirmed by the Lilliefors test. Student's *t*-test for paired samples was used for statistical comparison of the results. A *p* value < 0.05 was set as statistically significant.

## 3. Results

The mean temperature increase recorded on the mesial root surface was 7.5 ± 2.7°C (4.9–12.6), and the mean increase on the vestibular surface was 3.7 ± 2.1°C (1.3–7.8). The difference was highly significant (*p* ≈ 0).

**Figure 2:**
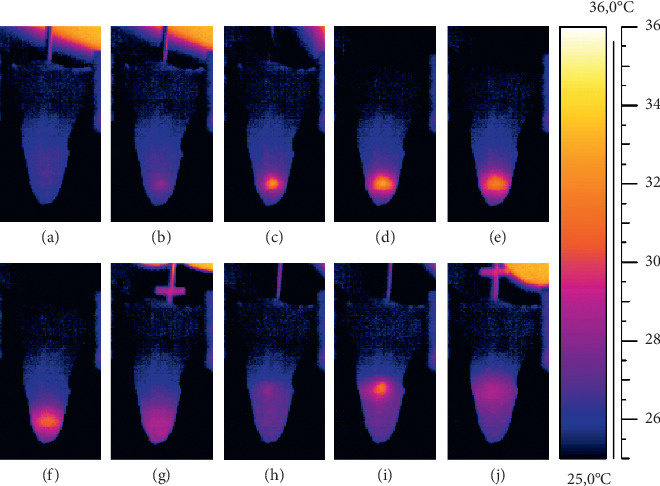
Thermograms from the mesial root surface of premolar teeth: (a, b, c) recorded during the first lasing; (d, e, f) recorded 1, 2, and 3 s, respectively, after the laser fiber removal; (g) recorded during vertical compaction of heated gutta-percha; (h, i) recorded during the second lasing; (j) recorded during second vertical compaction of heated gutta-percha.

**Figure 3:**
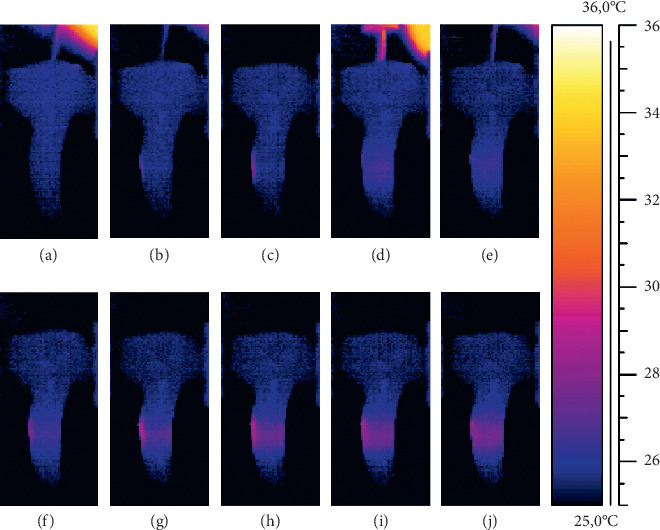
Thermograms from the vestibular root surface of premolar teeth: (a, b) recorded during the first lasing; (c) recorded 1 s after the laser fiber removal; (d) recorded during vertical compaction of heated gutta-percha; (e) recorded during the second lasing; (f, g, h, i, j) recorded 1, 2, 3, 4, and 5 s, respectively, after the laser fiber removal.

Figures 2 and 3 present the actual thermograms recorded during the root canal filling.

## 4. Discussion

Various types of lasers have been extensively studied in root canal treatments. Lasers have been investigated for their suitability in the preparation [18–22] and disinfection of the root canal [23–25] and in the removal of gutta-percha [26–28], but their usefulness for root canal filling has been investigated infrequently [16, 29, 30]. Maden et al. [29] compared conventional lateral condensation, Nd:YAG laser-softened gutta-percha, and the continuous wave of condensation technique using System B HeatSource to determine which caused the least apical leakage. Although there were no statistical differences among the three techniques, the System B group showed less leakage than the others, due to the creation of a homogeneous mass. Rocca et al. [30] used an Er:YAG laser to heat gutta-percha in order to create vertical condensation in the root canal space. They showed that the use of an Er:YAG fiber optic did not affect the quality of root canal fillings. However, the time required for filling was significantly less than that required for the conventional warm vertically condensed gutta-percha technique. Anić and Matsumoto [16] assessed the sealing ability of root canal fillings after either CO_2_ laser or Nd:YAG laser condensations of gutta-percha. In that study, apical sealing was evaluated with the dye penetration test. The most extensive dye penetration was observed in teeth obturated with composite resin, followed by gutta-percha lased with a CO_2_ laser and then gutta-percha lased with an Nd:YAG laser. The extent of dye penetration was the lowest in roots obturated with gutta-percha lased with an argon laser and condensed laterally and vertically. However, heat is generated during use of that laser; therefore, Anić and Matsumoto [12], in another in vitro study, assessed temperature increases on the tooth surface induced by laser-softened gutta-percha. The recorded temperature increases ranged from 12.9°C to 14.4°C. In the present study, we found lower temperature increases compared to those noted in [12]. This might be related to differences in study conditions, methods, and materials.

In this study, we found larger temperature increases on the mesial surface compared to the vestibular surface, probably due to the difference in the root wall thickness. Dentine is an excellent isolation material; thus, differences in root wall thickness can impact the heat transmitted. A root canal in the premolar teeth is flattened in the mesiodistal plane; therefore, both mesial and distal walls are much thinner than the vestibular and lingual walls [31]. In accordance with the findings of this study, the studies that evaluated the “continuous wave” obturation technique [32, 33] also found higher increases in temperature on the mesial root surface than on the vestibular surface. Furthermore, in a study of the ultrasonic removal of separated endodontic files without coolant, the outer root surface temperature changes were also found to be a function of root canal wall thickness [34].

Studies on temperature changes at the root canal surface following obturation with thermoplasticized gutta-percha have shown that the outer root surface temperature was influenced by the tooth type [32, 35]. This is related to the differences in root wall thickness among various types of teeth. In our study, the temperature was measured on the root surface of the premolar teeth. These teeth possess medium thickness root walls, i.e., thinner than maxillary incisors or canines, but thicker than mandibular incisors, buccal roots of maxillary molars, or mesial roots of mandibular molars. Therefore, it might be assumed that use of the laser in lower incisors would generate more heat on the external root surface than in premolars. However, that theory requires proper experimental proof. On the other hand, filling the root canals in upper incisors and canines is safe because those roots tend to be much thicker than roots of premolars [31].

The temperature values recorded in this in vitro study proved to be relatively low. The mean increase did not exceed 10°C; thus, the procedure should be considered safe. Additionally, it is possible that the in vivo temperature changes would be even lower because thermal energy dissipates more rapidly in vivo than in vitro. This is due to the circulation of blood in adjacent structures [36]. Furthermore, the thermal conductivity of vital tissues is different from that of air [37]. This difference might additionally influence the change in temperature. Venturi et al. [38] performed the continuous wave technique with artificial periodontal ligaments (alginate mass) and found notably lower temperature values than authors of studies that used air as the embedding medium. Similar results were obtained in studies on animal models. Endodontic procedures that generated a temperature increase of 22.3°C in an in vitro study did not produce any periodontal tissue damage in laboratory animals [39].

## 5. Conclusions

1. The results suggested that root canal filling with Nd:YAG laser-softened gutta-percha in premolar teeth should not damage the surrounding periradicular tissues.
2. To obtain valid temperature results, the measurement should be performed on the root surface with the thinnest wall.
3. Because temperature increase is related to dentin thickness, additional studies are required to assess the safety of laser procedures in teeth with thin root walls.

## Data Availability

The data used to support the findings of this study are available from the corresponding author upon request.

## Additional Points

(1) Nd:YAG lasers may be safe temperaturewise for plasticizing gutta-percha at the investigated parameters of 30 Hz frequency and a 200 mJ/pulse with optical fiber tips of 0.320 mm diameter. (2) Dentin has a significant effect in limiting the temperature rise generated during endodontic procedure. (3) To obtain valid temperature results, the measurement should be performed on the root surface with the thinnest wall.
